# Nutrition Practices for Healthy Living Across the Lifespan in Black/African American Men

**DOI:** 10.3390/nu17193127

**Published:** 2025-09-30

**Authors:** LLarance Turner, Chimene Castor, Oyonumo Ntekim

**Affiliations:** Department of Nutritional Sciences, Howard University, Washington, DC 20002, USA; oyonumo.ntekim@howard.edu

**Keywords:** Black men’s health, obesity in Black men, Black men’s nutrition, Black men’s lifestyle patterns

## Abstract

**Background:** Obesity is a well-established risk factor for numerous chronic diseases, including heart disease and type 2 diabetes, and its impact is particularly acute among Black/African American men. According to the Centers for Disease Control and Prevention (CDC), 70.9% of Black men aged 20 and older are overweight or obese. Despite this alarming prevalence, there remains a limited number of studies that specifically investigate the root causes of obesity in this population. Addressing this gap is critical to developing culturally relevant interventions that promote health equity. The purpose of this study was to assess dietary patterns, that are associated with overweight/obesity, in Black men aged 18–65 across the United States of America, to gain an in-depth understanding of variables influencing BMI in Black men. **Methods:** This study utilized a quantitative approach to collect information from participants. A survey questionnaire was developed and administered via Qualtrics to participants using a web link. The survey collected information across 18 dietary variables. Data was exported to Microsoft Excel for statistical analysis. A simple linear regression was used to determine dietary variables correlation strength and significance with BMI. A significance level of *p* < 0.05 was used to determine if a variable was statistically significant. Variables were then organized based on significance vs. non significance and correlation strength. **Result:** The study sample consisted of 466 Black men aged 18 to 65 years. The mean BMI was 30.21. Approximately 19% (*n* = 87) had a BMI within the healthy range (18.5–24.9), 41% (*n* = 190) were categorized as overweight (BMI 25.0–29.9), and another 41% (*n* = 189) were classified as obese (BMI ≥ 30.0). The findings revealed that fruit and vegetable consumption and whole grain cereal consumption were significantly and positively correlated with BMI. Other variables, such as fried foods, processed foods, and sugary drinks, though historically associated with obesity, did not show statistical significance in this population. **Conclusions:** Results suggest that while multiple dietary factors influence BMI, fruit, vegetable, and whole grain consumption are significantly correlated with BMI in Black men living in America. The findings from this study serve as a foundational step for designing targeted, culturally sensitive interventions aimed at reducing obesity-related health disparities. Future research should further explore how tailored public health messaging and community-based programming can address the specific needs of this population.

## 1. Introduction

Obesity remains a significant contributor to the global burden of non-communicable diseases and is especially prevalent among populations with longstanding health disparities. The World Health Organization (WHO) defines obesity as an excessive accumulation of body fat that presents a risk to health, operationalized in adults by a body mass index (BMI) of 30 kg/m^2^ or greater [1]. This clinical marker is strongly associated with adverse health outcomes, including cardiovascular disease, type 2 diabetes mellitus, dyslipidemia, and hypertension [2].

In the United States, the prevalence of obesity is disproportionately high among Black/African American men. According to the U.S. Department of Health and Human Services Office of Minority Health (OMH) in 2023 [3], the Centers for Disease Control and Prevention (CDC) reported 3.7% of non-Hispanic Black adults have been diagnosed with coronary heart disease, and 35.2% live with hypertension. Moreover, the CDC reported that between 2017 and 2020, 7.1% of non-Hispanic Black men have elevated cholesterol levels. Alarmingly, the CDC reported in 2021 a mortality rate of 246 per 100,000 non-Hispanic Black men attributable to heart disease [3]. Additionally, the CDC reported that between 2019 and 2021, 11.5% of Black men were diagnosed with diabetes, with a diabetes-specific death rate of 48.9 per 100,000. Data from the 2020 National Healthcare Quality and Disparities Report further highlight that 102.1 per 100,000 non-Hispanic Black adults experienced hospital admissions for uncontrolled diabetes without complications, while the National Healthcare Quality and Disparities 2020 reported 71.1 per 100,000 required lower-extremity amputations due to diabetes-related complications [4]. These statistics underscore a profound and persistent public health crisis requiring culturally informed, evidence-based strategies to mitigate obesity and reduce the burden of chronic disease in this population.

The etiology of obesity is multifactorial and includes biological, behavioral, environmental, and sociocultural determinants. Contributing factors include low socioeconomic status, inadequate nutrition, insufficient physical activity, limited sleep duration, chronic stress, systemic discrimination, and neighborhood disadvantage. Additionally, poor nutrition literacy, psychosocial stressors such as depression, and limited access to healthcare are interrelated risk factors that compound vulnerability to poor health outcomes [5,6].

Emerging literature has emphasized the critical role of dietary behaviors—specifically the overconsumption of energy-dense, nutrient-poor foods—in the pathogenesis of obesity. Diets high in ultra-processed foods, saturated fats, sodium, and added sugars, combined with insufficient intake of fiber-rich plant-based foods, are common among populations experiencing structural barriers to healthy living. Between 2015 and 2018, the CDC reported that 38.7% of African American/Black men aged 20 years and older were classified as obese, highlighting the magnitude of this epidemic [7].

Despite the known associations between dietary intake and chronic disease, there remains a limited quantity of research that examines obesity risk factors specific to Black men. This omission limits the development of tailored interventions. This study addresses this gap by examining the correlation between BMI and dietary intake across 18 dietary categories among Black men. The central hypothesis guiding this research is: The higher a Black man’s BMI, the more likely he is to exhibit lower intakes of fruits, vegetables, whole grains (made from complete grain kernel retaining bran, germ, and endosperm), and water, and higher intake of fried foods, ultra-processed foods (foods containing both additives and salt), meats, dairy, processed carbohydrates (carbohydrates altered during food production), sugary beverages, and added salt. The study also seeks to determine which dietary behaviors are most significantly associated with elevated BMI, thereby identifying key targets for culturally responsive obesity prevention strategies in Black/African American men.

## 2. Research Design and Overview of Methodology

This study employed a cross-sectional, quantitative research design to investigate the relationship between dietary behaviors and obesity among Black/African American men. A structured survey instrument was developed and administered to assess dietary consumption patterns, lifestyle habits, and potential correlates of elevated body mass index (BMI). The design was grounded in the principles of observational epidemiology, allowing for the identification of associations between dietary exposures and weight status in a real-world population.

Participants were asked to self-report their frequency of consumption across 18 distinct dietary categories, including fruits, vegetables, whole grains, processed foods, sugary beverages, meats, and added salt. The survey incorporated both closed- and open-ended questions to ensure comprehensiveness and ease of response, and was distributed using Qualtrics©, a secure online data collection platform. The tool integrated elements from validated dietary screeners, including the Alcohol Use Disorders Identification Test (AUDIT) and the “How Healthy is Your Diet?” questionnaire [8,9]. The instruments used in the study have well documented success rates and have been assessed via various research studies that target defined populations. Published literature shows that the instruments intended for use in the quantitative section of the study have been successfully applied internationally and translated into several languages thereby supporting their credibility and trustworthiness [10,11,12,13].

Data was collected from a convenience and purposive sample of 466 Black men aged 18–65 years. Multiple participants may have been diagnosed with a serious chronic disease (cancer; eating disorder, heart disease), currently or previously dealt with eating disorders, or have used or currently taking health medications. Participants with diagnosed mental impairments that would prevent accurate responses to the data instruments were excluded from the study. Recruitment strategies included social media outreach, community-based engagement, and participant referral. The sample size was determined based on a priori power analysis using G*Power 3.1 software, which indicated that a minimum of 262 participants would be required to achieve sufficient statistical power for linear regression analyses. A total of 466 responses were obtained, thereby exceeding the required threshold and enhancing the generalizability of findings.

Simple linear regression was employed to examine the strength and direction of associations between BMI (dependent variable) and each dietary behavior (independent variables). The regression models generated correlation coefficients (*r*), R-squared values, and significance tests (*p*-values) to assess whether dietary patterns were statistically linked to weight status. Since the type of test that was ran in the quantitative portion of this study is a Simple Linear Regression test, the “Test Family” was an “F tests”, the “Statistical Test” was “Linear multiple regression: Fixed Model, R^2^”, and the type of power analysis is “A priori: Compute required sample size—given *a*, power, and effect size”. The effect size *f*^2^, for this study is 0.05, the error of probability is 0.05, the Power is 0.95, and the number of predictors is 1. After inputting this into GPower 3.1 the total sample size needed to run a simple linear regression for this study was *n* = 262 participants. A significance level of *p* < 0.05 was used to determine meaningful associations. This study collected information from 466 participants to strengthen the accuracy of the research. The dietary variables were then ranked according to the strength of their correlation with BMI to identify the most influential behaviors.

This methodology provides a foundation for identifying culturally specific dietary patterns that may contribute to the disproportionate burden of obesity in Black men and highlights modifiable behaviors that can be addressed through targeted interventions.

### Study Information

The study was conducted according to the guidelines of the Declaration of Helsinki, and approved by the Institutional Review Board’s Office of Regulatory Research Compliance at Howard University. The IRB number of this study is IRB-2023-1240 and was approved on 17 September 2024. This research received no external funding. The authors declare no conflicts of interest. Informed consent was obtained from all participants involved in the study. Written informed consent has been obtained from the participants to publish this paper.

Participation in the study was voluntary, and all participants provided informed consent prior to completing the survey. Respondents were informed of the study’s purpose, their rights as participants, and the measures taken to ensure data confidentiality and anonymity. Written informed consent was obtained for both participation and publication of aggregate findings. This research was conducted without external funding, and the authors declare no conflicts of interest. The study team maintained full independence over the study design, data analysis, interpretation of findings, and manuscript preparation.

## 3. Results

This cross-sectional analysis examined the relationship between dietary behaviors and body mass index (BMI) among 466 Black/African American men aged 18 to 65 years. The average BMI in the study population was 30.21 kg/m^2^, categorizing the mean participant as obese. BMI categorization revealed that 19% of participants (*n* = 87) were within the healthy weight range (BMI 18.5–24.9), 41% (*n* = 190) were overweight (BMI 25.0–29.9), and another 41% (*n* = 189) met the clinical criteria for obesity (BMI ≥ 30.0).

**Figure 1:**
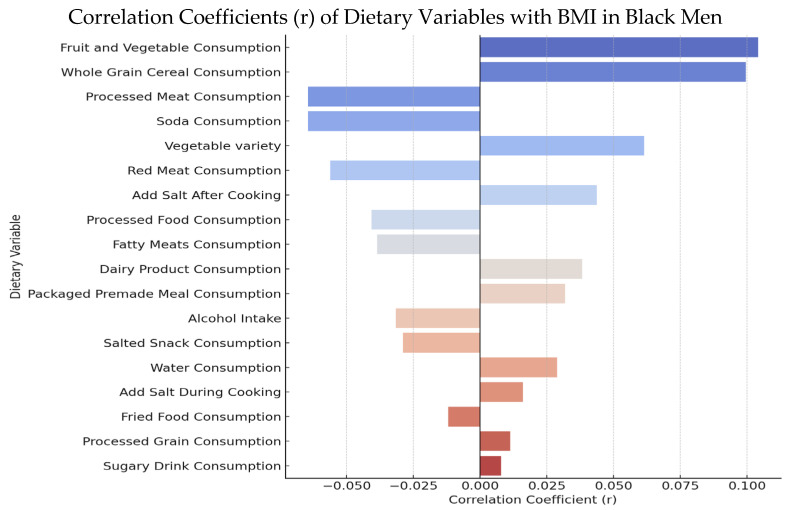
Correlation Coefficients of Dietary Variables with BMI in Black Men Visual showing strength and direction of linear correlations; fruit and vegetable intake and whole grain cereal consumption were statistically significant (*p* < 0.05).

Participants completed a comprehensive dietary survey capturing frequency of intake across 18 dietary variables. Responses were quantitatively analyzed using simple linear regression in Microsoft Excel to assess the strength and significance of association between each dietary variable and BMI. The resulting correlation coefficients (r) ranged from approximately −0.065 to 0.104. Figure 1 presents the dietary variables ranked by correlation strength.

### 3.1. Significant Dietary Correlates

Out of the 18 dietary categories evaluated, only two variables showed statistically significant associations with BMI:

1. Fruit and Vegetable Consumption (*r* = 0.1041, *p* = 0.0247)
2. Whole Grain Cereal Consumption (*r* = 0.0994, *p* = 0.0324)

Participants who reported consuming at least five servings of fruits and vegetables daily, and who regularly consumed whole grain cereals without added sugar (e.g., quinoa, barley, kamut, rye), exhibited lower mean BMI values than those with lower intake of these food groups. These findings are consistent with prior research demonstrating that higher intake of fiber-rich plant foods supports weight regulation and metabolic health [14,15].

### 3.2. Non-Significant Variables

Several other food groups, including processed meats, sugary drinks, red meats, fried foods, and dairy, did not demonstrate statistically significant correlations with BMI, though consumption patterns revealed behavioral trends. For example:

- Participants with a BMI ≥ 30 consumed more processed grains, sugar-sweetened beverages, and salted snacks than those with a BMI < 30.
- Dairy and fried food intake were slightly lower among participants with obesity, possibly reflecting dietary changes after diagnosis or underreporting.

These trends, though not statistically significant, suggest behavioral clustering that could inform targeted interventions.

### 3.3. Dietary Patterns in the Study Population

Table 1 provides a summary of dietary behaviors across the entire sample. Notably:

- Only 27% of participants regularly consumed whole grain cereals.
- Just 30% consumed at least five servings of fruits and vegetables per day.
- Water intake was adequate for 66% of respondents.
- High intake of processed food was observed in 76% of the population.
- Frequent consumption of red meat (40%), fatty meat (49%), and sugary drinks (51%) was also reported.

In contrast, low intake was observed for processed meats, soda, salt added after cooking, and pre-prepared packaged meals, which may reflect either dietary preferences or access limitations.

### 3.4. Interpretation

The data reflect suboptimal dietary patterns in this population, characterized by low intake of protective foods (e.g., fruits, vegetables, whole grains) and moderate-to-high intake of risk-associated foods (e.g., processed foods, sugary beverages). Although most dietary behaviors were not significantly correlated with BMI at the individual variable level, the cumulative pattern suggests elevated nutritional risk.

The significant associations found for fruit, vegetable, and whole grain intake affirm their role in BMI regulation and support prioritizing these behaviors in public health interventions targeting obesity in Black men.

## 4. Discussion

Numerous studies have demonstrated that poor dietary behaviors are central to the development of obesity and its associated comorbidities, such as cardiovascular disease, type 2 diabetes, and metabolic syndrome. Among the many determinants of chronic illness, dietary patterns have consistently emerged as a modifiable risk factor with profound implications for long-term health outcomes, particularly in vulnerable populations. In this study, we investigated how various dietary behaviors correlate with BMI among a sample of 466 Black/African American men—a demographic disproportionately burdened by obesity and chronic diseases.

Consistent with national data, a significant proportion (82%) of the study population was classified as overweight or obese. Regression analysis revealed that fruit and vegetable consumption and whole grain cereal intake were the only dietary variables significantly and positively associated with BMI. Participants with a BMI ≥ 30 reported lower intake of fruits, vegetables, and whole grains compared to those with a BMI < 30, aligning with evidence from previous studies indicating that insufficient consumption of fiber-rich plant-based foods contributes to excess body weight and increased cardiometabolic risk.

Notably, some traditionally cited contributors to obesity—such as fried foods, sugary beverages, and ultra-processed items—did not exhibit statistically significant associations with BMI in this sample, despite trends suggesting higher frequency of intake among participants with obesity [16,17,18,19]. For instance, men with a BMI ≥ 30 reported greater consumption of processed foods, sugary drinks, red meats, and fatty meats. Variables that were statistically proven to be non-significant is due to the limited quantity of the food item consumed based on survey instruments, and not due to the food item not being consumed at all. Expected significant risk factors mentioned in hypothesis (e.g., fried foods, ultra-processed foods) could have potentially not reached significance since obesity can be multi-factorial, thus other variables including measurement limitations of food items, physical activity levels, food desserts, calorie consumption levels, family history, or other variables could play a part in BMI range of participants. Although these associations did not reach the threshold for statistical significance, suggesting that while these foods may still play a role in long-term weight gain and disease development, they may not be the most immediate predictors of elevated BMI in this population. Further in depth studies (quantitative and qualitative) are needed to determine why foods that are recognized for being associated with obesity did not affect this population’s obesity rate significantly.

Interestingly, water consumption was higher among participants with obesity, which may be reflective of greater health consciousness among those already affected by weight-related health concerns or a compensatory behavior due to dietary imbalances. Similarly, the observation that men with higher BMI consumed fried foods less frequently than those with lower BMI may point to underreporting bias or recent dietary modifications prompted by medical advice or diagnosis.

This study does not discount the importance of ultra-processed foods and high-sodium intake in obesity pathology; rather, it suggests that these variables may exert their influence in complex, context-dependent ways. It is also possible that the lack of statistical significance may be due to measurement limitations, including self-reported intake and the categorical rather than continuous nature of dietary frequency data. Furthermore, the consumption of salted snacks, red meats, and processed grains was higher among participants with obesity, suggesting a clustering of behaviors that may contribute to cumulative risk, even if not individually significant. Importantly, the reduced intake of dairy products and increased intake of processed meats among obese participants may reflect patterns of food substitution and cultural dietary preferences that merit further exploration.

### 4.1. Strengths and Limitations

A key strength of this study is the relatively large and demographically focused sample of Black/African American men—an understudied population in obesity-related dietary research. The use of validated survey instruments and a comprehensive dietary behavior framework enhances the reliability of the data. By isolating 18 dietary categories, the study provides granular insights into specific behaviors that may serve as focal points for intervention.

However, the study is not without limitations. First, the cross-sectional design limits causal inference. Second, all dietary data were self-reported, which introduces the potential for recall bias and social desirability bias. Third, the survey did not capture total caloric intake, macronutrient composition, or meal timing, which are all known to influence BMI. Additionally, the lack of biochemical markers and anthropometric measures beyond BMI limits the physiological interpretation of findings. This study’s convenience sample limits generalizability to the entire population of black men. This study also included self-reporting which can pose a risk of underestimating weight, overestimating height and recall/reporting bias in food consumption. Self-reporting dietary intake may contribute to the lack of significant associations in certain food groups. Furthermore, the cross-sectional design prevents causality from being established, whether food consumption influences BMI or whether BMI influences consumption patterns. This study also does not account for confounding factors (e.g., physical activity levels, socioeconomic status). Despite these limitations, the study lays a critical foundation for future investigations. It emphasizes the need for culturally relevant, behaviorally targeted obesity interventions for Black men. The identification of fruit and vegetable consumption and whole grain intake as significant predictors of BMI should inform the design of public health messaging and community-based nutrition education programs.

Future studies should employ longitudinal methods, incorporate more precise dietary assessment tools (e.g., 24-h dietary recalls or food diaries), and explore psychosocial and environmental mediators of eating behaviors. The integration of biological markers would also strengthen the validity of dietary–biomarker–disease relationships in this population.

### 4.2. Implications

The implications of this study suggest a targeted focus on increasing consumption of fruits, vegetables, and whole grains to combat obesity in Black men. Public health initiatives should incorporate culturally tailored nutrition education and community-based interventions. Faith-based organizations, local farms, and barbershop-based health programs could be leveraged as trusted avenues to deliver these messages. Further, healthcare providers should receive training to understand the cultural factors influencing dietary choices in Black communities.

## 5. Conclusions

Obesity is highly associated with the development of multiple chronic diseases including heart disease and type 2 diabetes. Behavioral patterns that contribute to the high rate of obesity among Black men in the United States is under researched and warrants attention. This study focused on multiple dietary categories that have been proven to be correlated with obesity in all Americans. The purpose of this study was to determine which of these variables were significantly correlated in Black men. The information gathered in this study supported the hypothesis that Black men with higher BMI had lower consumption of fruits, vegetables, and whole grains compared to Black men with a normal range BMI, but did not support the hypothesis that Black men with an overweight/obese BMI status consumed a higher intake of fried foods, ultra-processed foods, meats, dairy, processed carbohydrates, sugary beverages, and added salt compared to Black men with a normal range BMI status. Various social determinants of health and other factors that determine BMI status (physical activity levels, family history, medications prescribed, mental health status, and other variables) would need to be further researched in this population to strengthen the understanding of the overweight/obesity rates in Black men.

The information gained from this study demonstrates where the primary focus should be placed when devising future strategies to develop dietary interventions to reduce obesity rates among Black men and can be very useful in crafting messages to the Black male community.

## Figures and Tables

**Table 1:** Dietary Patterns Among Black Men (*N* = 466).

Dietary Behavior	Yes (*n*)	No (*n*)	% Yes
Regular Whole Grain Cereal	123	340	27%
≥5 Fruit and Vegetable Servings	138	327	30%
≥4 Vegetable Varieties/Week	244	221	52%
Fried Food ≥ 2×/week	215	251	44%
Processed Food ≥ 2×/week	355	111	76%
Dairy ≥ 3×/week	220	247	47%
Fatty Meat in ≥ 3 Meals/Week	227	236	49%
Red Meat in ≥ 3 Meals/Week	187	277	40%
Processed Meat ≥ 2×/week	139	325	30%
Processed Grains Frequently	359	108	77%
Sugary Drinks ≥ 2×/week	240	227	51%
Soda ≥ 1×/week	157	310	34%
Adds Salt During Cooking	317	150	68%
Adds Salt at Table	101	366	22%
Salted Snacks ≥ 2×/week	248	219	53%
Adequate Water Intake	157	307	34%
Eats Pre-Prepared Meals	73	394	16%
Alcohol ≥ 4×/week	94	372	25%

## Data Availability

The data supporting this study’s findings are available from Howard University. Access to the data can be requested from the corresponding author upon reasonable request with the permission of Howard University. They are not publicly available due to privacy and ethical restrictions.
